# Knowledge and attitudes towards antibiotics and antimicrobial resistance among patients in rural South Africa and the implications for future policies

**DOI:** 10.3389/fphar.2026.1756239

**Published:** 2026-04-10

**Authors:** Tiyani Milta Maluleke, Morgan Tiyiselani Maluleke, Nishana Ramdas, Ana Golić Jelić, Amanj Kurdi, Inaam Ur Rehman, Stephen M. Campbell, Vanda Marković-Peković, Natalie Schellack, Audrey Chigome, Aislinn Cook, Brian Godman, Johanna C. Meyer

**Affiliations:** 1 Department of Public Health Pharmacy and Management, School of Pharmacy, Sefako Makgatho Health Sciences University, Garankuwa, Pretoria, South Africa; 2 Saselamani Pharmacy, Saselamani, South Africa; 3 Department of Pharmacy, Faculty of Medicine, University of Banja Luka, Banja Luka, Bosnia and Herzegovina; 4 Strathclyde Institute of Pharmacy and Biomedical Sciences, Strathclyde University, Glasgow, United Kingdom; 5 College of Pharmacy, Hawler Medical University, Erbil, Iraq; 6 College of Pharmacy, Al-Kitab University, Kirkuk, Iraq; 7 Yashfeen College of Pharmacy, Yashfeen Education System, Lahore, Pakistan; 8 School of Health Sciences, University of Manchester, Manchester, United Kingdom; 9 Department of Pharmacology, Faculty of Health Sciences, University of Pretoria, Pretoria, South Africa; 10 South African Vaccination and Immunisation Centre, Sefako Makgatho Health Sciences University, Garankuwa, Pretoria, South Africa; 11 Antibiotic Policy Group, Institute for Infection and Immunity, City St. George’s University of London, London, United Kingdom; 12 Nuffield Department of Primary Care Health Sciences, University of Oxford, Oxford, United Kingdom

**Keywords:** antibiotics, antimicrobial resistance, attitudes, community pharmacies, health policies, language, patients, South Africa

## Abstract

**Background:**

Antimicrobial resistance (AMR) is an appreciable public health threat, exacerbated by considerable inappropriate use of antibiotics including for upper respiratory tract infections (URTIs). Whilst there have been high levels of inappropriate prescribing of antibiotics in primary care in South Africa, study findings vary regarding the extent of dispensing of antibiotics without a prescription. Where this occurs, this is typically for patients with urinary tract infections (UTIs) and sexually transmitted infections (STIs). Consequently, there is a need to update knowledge regarding antibiotic dispensing patterns in primary care in South Africa alongside key factors influencing this. The findings can provide future direction to key stakeholders in South Africa grappling with high AMR rates.

**Methods:**

A previously piloted questionnaire was administered to patients leaving community pharmacies in a rural province using their preferred language. The questionnaire collected data on current antibiotic utilisation patterns alongside their knowledge and attitudes towards AMR.

**Results:**

465 patients were interviewed exiting community pharmacies with a medicine. 78.7% of patients who were dispensed antibiotics were dispensed these without a prescription. Perceived STIs were the most common infectious disease where this occurred, with 99.1% of antibiotics issued for this condition dispensed without a prescription. Only 1 out of 116 patients with a perceived STI, received an antibiotic from a prescription issued by an authorized prescriber. The reverse was seen with patients with URTIs where there was very little dispensing of antibiotics without a prescription for these patients. This may be because surveyed patients were prepared to take advice from community pharmacists, who typically offered symptomatic relief to patients with suspected URTIs. This situation contrasts with antibiotics from prescriptions where URTIs were the most common infection where antibiotics were prescribed (59.3%). Questioning patients in their own language enhanced their understanding of key issues.

**Conclusion:**

There is an urgent need to re-consider community pharmacist activities in South Africa with some countries allowing them to prescribe antibiotics for UTIs. Trained community pharmacists can also potentially engage with patients to help prevent and manage STIs with patients appearing to preferentially seek assistance from community pharmacists for their perceived STIs. Community pharmacists can also potentially work with prescribers to improve their antibiotic use especially for URTIs.

## 1 Introduction

Bacterial antimicrobial resistance (AMR) is increasing globally, with AMR rapidly becoming the next pandemic (The Lancet, 2024; Patra et al., 2025). Bacterial infections are now the second leading cause of death globally (The Lancet, 2024), with published estimates suggesting there were up to 5 million deaths globally in 2019 alone associated with bacterial resistance (Murray et al., 2022). The greatest burden of AMR, and associated mortality, is currently seen among low- and middle-income countries (LMICs), which includes sub-Saharan Africa (Murray et al., 2022; Antimicrobial Resistance Collaborators, 2024). As a result, AMR is now the leading cause of death in Africa (Antimicrobial Resistance Collaborators, 2024). It is projected that by 2050 there could be up to 4.1 million AMR-related deaths in sub-Saharan Africa alone unless urgent actions are undertaken (Totaro et al., 2025). There are also considerable economic and resource implications associated with AMR among LMICs (The Lancet, 2024).

Inappropriate antibiotic use is a key driver of AMR along with their growing utilisation, with antibiotic use increasing by 65% globally from 2000 to 2015 (Klein et al., 2024; Abejew et al., 2024; Wang et al., 2025). The increasing use of Watch and Reserve antibiotics among LMICs, with their greater resistance potential, is further adding to AMR (Klein et al., 2021; Klein et al., 2024; Al Masud et al., 2024; ESCMID, 2025).

South Africa is similar to other LMICs with high rates of AMR, enhanced by antibiotic use increasing by 50% in the public sector between 2018 and 2022 (Antimicrobial Resistance Collaborators, 2024; Department of Health Republic of South Africa, 2024; Finlayson et al., 2024; Founou et al., 2024; Ahmed et al., 2025; Hetsa et al., 2025). High rates of AMR in South Africa is a concern, considering a decrease in the use of Access antibiotics in the public sector, down to 48% of total antibiotic use in 2022, with a corresponding increase in the use of Watch antibiotics (Department of Health Republic of South Africa, 2024). Alongside this, there are growing concerns with sub-optimal adherence to current antibiotic guidelines among prescribers in South Africa, further increasing AMR (Chigome et al., 2023; Department of Health Republic of South Africa, 2024).

Another key challenge among LMICs when trying to reduce AMR is addressing current high levels of inappropriate dispensing of antibiotics without a prescription, including Watch and Reserve antibiotics, for essentially self-limiting conditions (Li et al., 2023; Gashaw et al., 2025; Saleem et al., 2025; Zewdie et al., 2025). The principal reasons for this activity include the accessibility of community pharmacies compared with the challenges involved with accessing healthcare professionals (HCPs) in primary healthcare (PHC) facilities, as well as any associated travel costs, often long-waiting times in PHC facilities, co-payments when seeing HCPs, beliefs among patients that their infectious diseases are only minor and consequently not worth visiting PHC facilities, as well as patient demands for antibiotics often exacerbated by their limited knowledge regarding antibiotics, AMR and antimicrobial stewardship (AMS) (Torres et al., 2019; Li et al., 2023; Gashaw et al., 2025; Kapatsa et al., 2025). Limited knowledge of key issues surrounding antibiotics, AMR, and AMS, among patients is a major concern across LMICs adding to AMR when combined with their increasing influence on both prescribers and dispensers of antibiotics (Supplementary Tables S1 and S2). This is a key issue among LMICs since antibiotic use in primary care in these countries can account for up to 90% of total antibiotic use among patients (Duffy et al., 2018; WHO, 2025). Alongside this, there has been no real change in the rate of dispensing of antibiotics without a prescription among LMICs in recent years despite a number of initiatives (Li et al., 2023; Saleem et al., 2025).

There has been contradictory evidence regarding the extent of purchasing of antibiotics without a prescription in South Africa in recent years despite this activity currently being prohibited (Anstey Watkins et al., 2019; Do et al., 2021; Mokwele et al., 2022; Sono et al., 2024a). Published studies by Anstey Watkins et al. (2019) and Do et al. (2021) showed little or no purchasing of antibiotics without a prescription among rural communities in South Africa (Anstey Watkins et al., 2019; Do et al., 2021). This situation may have been mitigated by the fact that there is universal healthcare (UHC) in South Africa; consequently, no co-payment if medicines are dispensed in PHC facilities in these rural communities (Meyer et al., 2017; Do et al., 2021). This contrasts with the findings of Mokwele et al. (2022), who documented rates of 80% of purchasing antibiotics without a prescription among simulated patients in privately owned pharmacies in principally urban settings; however, there was no purchasing of antibiotics without a prescription among participating chain pharmacies (Mokwele et al., 2022). There have also been variable findings regarding the extent of dispensing of antibiotics without a prescription in the studies of Sono et al. (2024a), Sono et al. (2024b), Sono et al. (2025), and Maluleke et al. (2025a). In the study of Maluleke et al. (2025a) only an estimated 8.6% of the total number of antibiotics dispensed from rural community pharmacies were dispensed without a prescription (Maluleke et al., 2025a). However higher rates of dispensing were seen when patients were surveyed in two pilot studies in the same rural province (Sono et al., 2024b; Sono et al., 2025).

Consequently, in view of ongoing controversies and concerns in South Africa regarding both the prescribing and dispensing of antibiotics in primary care (Supplementary Table S3), including the increasing utilisation of Watch and Reserve antibiotics (Chigome et al., 2023; Department of Health Republic of South Africa, 2024; Sono et al., 2024a; Chigome et al., 2025; Maluleke et al., 2025a), as well as issues surrounding the influence of patients on subsequent antibiotic use among LMICs (Supplementary Table S1), we believe there was a need to build on recent studies to help provide guidance to key stakeholder groups in South Africa. This includes further studies questioning patients regarding any purchasing of antibiotics without a prescription, their rationale for this activity, as well as their knowledge of, and attitudes towards, key aspects of antibiotics and AMR, The additional motivation for interviewing patients is because we are aware from published studies that community pharmacists and pharmacist assistants may under-report the extent of their selling of antibiotics without a prescription, especially if such activities are currently prohibited (Edessa et al., 2022; Sono et al., 2024a; Maluleke et al., 2025b). In addition, we are aware that there can be challenges among patients with their understanding of key terms, including antibiotics and AMR, especially if the pharmacist or pharmacist assistant is not speaking to them in their native language and these terms are not readily translatable (Anstey Watkins et al., 2019; Mokoena et al., 2021; Sono et al., 2024b; Sono et al., 2025). Consequently, we also wanted to explore this further.

As a result, the primary focus of this study was to assess patients’ knowledge and attitudes towards antibiotics and AMR as well as whether there is any link between their knowledge of key issues surrounding antibiotics, AMR, and AMS and their requests for antibiotics without a prescription. The secondary focus is to assess the extent of antibiotics being dispensed without a prescription among patients exiting community pharmacies, their perceived infection, and the rationale for this activity, alongside issues of language. The key analytical contribution lies in linking antibiotic dispensing patterns with or without a prescription to patients’ understanding and attitudes towards antibiotics, AMR and AMS, to guide future stewardship interventions given increasing concerns with rising AMR rates in the country (Mendelson, 2022; Mendelson et al., 2025a).

## 2 Methodology

### 2.1 Study design

This study formed part of a larger mixed-methods project, and employed a cross-sectional descriptive survey among patients exiting community pharmacies in a rural South African province (Maluleke et al., 2025c). The province was purposively selected due to well-documented barriers to accessing PHC facilities in this province, which included long travel distances, extended waiting times, and physician shortages (Ntuli and Maboya, 2017; Maluleke et al., 2025a; Maluleke et al., 2025c; Maluleke et al., 2025d; Mendelson et al., 2025b). These challenges increase the likelihood that patients will first seek care at community pharmacies where possible to manage their conditions including infectious diseases. This can potentially include patients trying to purchase antibiotics without a prescription directly from community pharmacies, especially if they believe their disease resembles a previous episode successfully treated with antibiotics, including for self-limiting viral infections, and/or if they consider their condition as minor and not worth the time, effort and costs to visit a HCP in a PHC facility (Li et al., 2023; Sono et al., 2023; Sono et al., 2024a).

This study was intentionally designed to collect data directly from patients immediately after they exited community pharmacies. As a result, allowing information to be directly and speedily captured from potential participants. This avoided the possibility of low inclusion rates if patients were approached in other settings. In addition, possible forgetfulness of key information, which could include the nature and extent of any antibiotics dispensed with a prescription or purchased, the indication for any antibiotics dispensed and their rationale, if the interviews were conducted some time later. By interviewing patients directly as they exited community pharmacies, the interviewers were also able to view directly which antibiotics had been dispensed as well as ask the perceived corresponding infectious disease for any antibiotics dispensed (Maluleke et al., 2025c). In addition, try as far as possible to interview patients in the same locality as the survey among with community pharmacy personnel as part of the larger study to be able to compare and contrast the findings (Maluleke et al., 2025a; Maluleke et al., 2025d).

### 2.2 Target population and study sample

Community pharmacies in South Africa are principally categorised into three groups, with the majority of community pharmacies being independent pharmacies, followed by franchise and chain pharmacies (Maluleke et al., 2025a). The target population for this study were patients who exited community pharmacies across the province having bought their medicines, which in the first instance included antibiotics without a prescription or over-the-counter (OTC) medicines, or had their medicines dispensed following a prescription including antibiotics. OTC medicines were included as we were aware from the pilot study with community pharmacy personnel that they mostly or always offered symptomatic treatment for patients presenting with self-limited symptoms rather than suggesting antibiotics (Sono et al., 2024a).

A minimum sample size for a descriptive survey was calculated at 385, considering a target population of 900,106 adults accessing private healthcare in the province (Ntuli and Maboya, 2017), and assuming a response distribution of 50% to give the largest sample size at 90% power and 95% confidence level (Epi Info version 7.2.6.0; Centers for Disease Control and Prevention, Atlanta, United States). The target sample size was increased to 420 patients to allow for any incomplete data.

Inclusion criteria for participation were adults aged 18 years and older exiting community pharmacies with at least one medication, including prescribed or non-prescribed antibiotics or OTC medicines, and capable of providing informed consent. Patients not carrying any medication were excluded, as the study aimed to evaluate knowledge and behavior among medicine users. Patients unwilling or unable to provide consent were also excluded from participation (Sono et al., 2024b; Sono et al., 2025). Considering the feasibility, available resources and practical implications, and given the landscape of community pharmacies in this rural province, a convenient sampling strategy was used to select patients for an interview as they exited community pharmacies whilst also aiming for a patient sample that reflected the proportional representation of the different pharmacy categories in the province (Table 1). As mentioned, the objective being to enhance the contextual validity of the findings as well as ensuring coverage of the varying regulatory and operational frameworks common to these pharmacy outlets in rural and peri-urban communities. Table 1 provides further details of the patient sample size, distributed by the different community pharmacy categories in this province.

**TABLE 1:** Categories of community pharmacies in a rural South African province (adapted from Maluleke et al. (2025a)) and the targeted sample of patients for recruitment.

Pharmacy category	Community pharmacy category description	Number* (%) of pharmacies in the province	Proportional number (%) of patients targeted for recruitment
Chain pharmacies	Pharmacies are owned by corporate entities, such as Clicks, Dischem and ‘Medirite’ at Checkers (supermarket). Operations are managed under a centralized system with standardized store branding.	38 (14.0%)	59 (14.0%)
Franchise pharmacies	Independently owned pharmacies by franchisees; however, operations are under a common brand name. Examples include ‘Link’, ‘The Local Choice’ and ‘Van Heerden’.	71 (26.1%)	110 (26.2%)
Independent pharmacies	Standalone pharmacies with no corporate or franchise affiliations. Their clientele, size and business models vary across the province.	163 (59.9%)	251 (59.8%)
Total		272	420

NB: *Numbers are based on the publicly accessible pharmacy register at the time of the study (https://interns.pharma.mm3.co.za/SearchRegister). Six pharmacies in the province were non-operational at the time of the study, hence not included in the target population.

### 2.3 Patient questionnaire development

The patient questionnaire (Supplementary Table S4) was specifically designed for the purpose of this study. It was based on previously published studies combined with input from key personnel in this area, which included leading academic personnel researching key aspects of antibiotics and AMR in South Africa and wider (Kretchy et al., 2021; Mokoena et al., 2021; Darshansinh et al., 2023; Nguyen et al., 2023; Sono et al., 2023). Variables contained in the questionnaire included the kind of medicines being purchased, or dispensed, including antibiotics, whether the antibiotics dispensed were with or without a prescription, the perceived infectious disease for which patients sought antibiotics, as well as patients’ knowledge of, and attitudes towards, antibiotics and AMR (Maluleke et al., 2025c).

Pilot studies had previously been conducted to evaluate the suitability of the patient questionnaire (Sono et al., 2024b; Sono et al., 2025), with the pilot-testing focussed on a number of key issues. These included the clarity, contents, face validity, and contextual appropriateness of the questions and methodology. The pilot questionnaire was interviewer-administered followed by direct feedback from patients. Patient feedback was used to modify the questionnaire where pertinent as well as address any potential shortcomings and challenges encountered during the pilot studies, especially with the questionnaire being translated from English into three local languages (Sono et al., 2024b; Sono et al., 2025). The goal was to ensure that the instrument used in the main study could successfully elicit the necessary information to meet the study objectives, with the combined approach similar to other published studies in this area among LMICs (Mekuria et al., 2019; Sindato et al., 2020; Acharya et al., 2021; Ndaki et al., 2023; Altaye et al., 2024).

The final questionnaire was divided into two parts (Supplementary Table S4). Part 1 collected information on patients’ sex, their educational level, the nature of the community pharmacy they exited from, the kind of medicines dispensed or sold, whether these included an antibiotic, whether the antibiotics were dispensed with a prescription or not, and the indications or conditions mentioned by patients for which they sought treatment. No attempt was made to ask the patient about their current occupation as the key concerns were issues of understanding and health literacy given generally poor knowledge regarding antibiotics and AMR seen among patients in LMICs (Supplementary Table S1). Potential indications for antibiotic dispensing, based on previous publications and the pilot studies with both community pharmacists, pharmacist assistants and patients, included URTIs, STIs, UTIs, and skin and soft tissue infections (SSTIs) (Godman et al., 2020; Mokoena et al., 2021; Sono et al., 2023; Sono et al., 2024a; Sono et al., 2025).

No attempt was also made to check the patients’ infectious disease by discussing with them their symptoms, and any associated discussions between them and any HCP, as this was not the aim of the study. In addition, it was considered too intrusive to discuss the actual symptoms with patients. Consequently, the symptoms for each antibiotic dispensed were based purely on the replies from patients acknowledging the potential for misclassification bias this entails. The perceived indications for the antibiotics dispensed covered both those dispensed with a prescription and those purchased without a prescription. Following this, the patient’s rationale for purchasing antibiotics without a prescription was elicited alongside the key reasons for approaching particular community pharmacies in the first place.

The objective of Part 2 of the questionnaire, which is the principal focus of this paper, was to evaluate patients’ knowledge regarding key aspects of antibiotics and AMR (Sono et al., 2024b). In addition, their attitudes towards potential ways forward to help reduce AMR in South Africa. The questionnaire included ten knowledge statements and five attitude statements, with each statement having three response options, namely, ‘True’, ‘False’ or ‘Don’t know’ (Kandasamy et al., 2020; Orubu et al., 2022). Five of the ten knowledge statements, and one of the five attitude statements, were negatively worded in order to reduce response bias, particularly acquiescence bias. Consequently, helping to prevent patients from simply agreeing with every item and forcing them to read, or listen to, each statement carefully, before responding.

Knowledge and attitude scores were categorized using established cut-offs widely applied in antimicrobial use and knowledge, attitude and practice (KAP) surveys among LMICs (Andrade et al., 2020; Sefah et al., 2022; Saleem et al., 2025). Knowledge scores were categorized based on prior validated thresholds shown to discriminate patient understanding effectively in similar LMIC contexts. Attitude scores were similarly categorized reflecting common methods to assess patient perspectives in stewardship research (Sefah et al., 2022; Saleem et al., 2025). We chose this approach as we believe this facilitates comparability with other KAP studies regarding antibiotic use, AMR and AMS (Saleem et al., 2025).

Negatively worded statements were reverse scored during data analysis to maintain consistency, which ensured that high scores consistently reflected high levels of their measured knowledge and attitudes across all items regardless of whether they were positively or negatively worded. The final statements were also phrased in a deliberately direct manner to minimise ambiguity particularly during translation. This approach to the questionnaire development, with combined insights from published literature, input from experts and subsequent pilot testing, reflects the methodology commonly reported in several published studies in this field (Mate et al., 2019; Gacheri et al., 2024; Darweesh et al., 2025).

The final questionnaire was translated into three commonly spoken local languages, namely, Sepedi, Tshivenda and Xitsonga, to facilitate patient understanding of key terms included in the questions (Sono et al., 2025). Translation was conducted by linguistic experts who were fluent in both English and the targeted local languages, as well as being very familiar with the terminology (Sono et al., 2024b; Sono et al., 2025). This is similar to the approach of Mokoena et al. (2021) where the study questionnaire was translated into IsiZulu and Setswana as these were the local languages spoken most frequently by the surveyed taxi community (Mokoena et al., 2021).

### 2.4 Patient recruitment and data collection

Trained research assistants collected data through face-to-face interviews with patients immediately after exiting community pharmacies across this rural province. The data collectors positioned themselves in the vicinity of pharmacy entrances and approached individuals carrying medicine bags or pharmacy shopping bags.

Patients were recruited independently of pharmacists and pharmacist assistants, who had participated in a separate survey as part of the larger project (Maluleke et al., 2025d), in order to avoid bias or overlap between the two surveys conducted concurrently. However, enhance the contextual validity of the findings. Only patients who had purchased medicines, or had these dispensed, and provided voluntary consent, were included in the study. Data collectors remained in the vicinity of the community pharmacy for approximately two hours before moving to another site to ensure coverage across the different pharmacy categories and across this rural province.

Study objectives were explained to prospective participants, and an information sheet was read aloud in English, Xitsonga, Tshivenda or Sepedi. Written informed consent was subsequently obtained, with the consent forms available in all four languages. Interviews were conducted in the participant’s preferred language and no identifiable information was collected (Supplementary Table S4), ensuring anonymity and confidentiality.

In addition to administering the questionnaire, data collectors also recorded field notes on patients’ reactions and questionnaire clarity during the interview. These notes were reviewed to identify potential recurring themes related to language comprehension, and its influence, regarding their understanding of antibiotics and AMR. As a result, providing contextual depth to the findings and any informed recommendations for improving future patient-provider communications as well as future education strategies.

### 2.5 Data management and analysis

The data entry process was conducted using Microsoft Excel™, with TMM capturing all responses from the completed questionnaires. This was followed by one of the research assistants performing a double-check of the data entry to ensure accuracy and reliability of the data entry. Any discrepancies identified during this verification process were resolved through cross-checking against the raw paper-based questionnaires used to capture responses. The data was then cleaned and coded to standardize categorical responses, and subsequently prepare variables for statistical analysis. The finalized dataset was imported into Jamovi (https://www.jamovi.org/) for statistical analysis. Descriptive statistics were used to summarize the data, with frequencies and percentages calculated for categorical variables, and means with standard deviation (SD), alongside medians with interquartile ranges (IQR), for continuous variables.

In terms of accessing antibiotics, patients were principally categorised into those who had purchased antibiotics without a prescription versus those who received antibiotics with a prescription as this was the key group for this study. Patients with medicines included those with OTC medicines since all patients who had a medicine or pharmacy bag were approached for possible interviews. In addition, we wanted to include those patients who had been dispensed symptomatic relief for their self-limiting infections. This was because community pharmacy personnel had previously stated that they always, or mostly offered, symptomatic relief to these patients rather than recommending antibiotics (Sono et al., 2024a).

For the ten knowledge statements, each correct answer was scored 1, while incorrect answers, including ‘Don’t know’, were scored as 0. The five negatively worded knowledge statements were reverse scored, after which a total score for each patient was calculated. The maximum total possible score was 10, ranging from 0 to 10, with a higher total score indicating better knowledge. Knowledge scores were also converted to categorical data using cutoffs, namely, ‘Poor knowledge’ (score: 0–4), ‘Average knowledge’ (score: 5–8), and ‘Good knowledge’ (score: 9–10). For the five attitude statements, each positive answer was scored 1, while negative answers, including ‘Don’t know’ were scored 0. One negatively phrased statement was reverse scored, and a total attitude score was subsequently calculated for each patient. Attitude scores ranged from 0 to 5, with 5 being the maximum possible score and higher scores indicating a positive attitude. Attitude scores were also converted to categorical data using cutoffs, namely, ‘Negative attitude’ (score: 0–2), ‘Neutral attitude’ (score: 3), and ‘Positive attitude’ (score: 4–5). Overall, the scoring and categorization of knowledge and attitude items in this study were based on approaches used in previous knowledge, attitude and practice (KAP) surveys across a range of disease areas, where correct/positive responses were scored as 1, incorrect/negative/‘Don’t know’ responses as 0, and negatively worded items were reverse scored (Andrade et al., 2020; Sefah et al., 2022).

Inferential analysis was used to measure associations between patients’ knowledge of, and attitudes toward, the use of antibiotics and AMR, and purchasing antibiotics without a prescription. Statistics included calculation of odds ratios (ORs) with the 95% CI around the OR, Chi-square (χ^2^) or Fisher’s exact tests as appropriate and the Student’s t-test or One-way ANOVA for comparison of mean scores. Statistical significance was set at p < 0.05.

### 2.6 Ethical approval

Ethical approval for the study was obtained from the Sefako Makgatho University Research Ethics Committee (SMUREC/P/229/2023:PG). Upon invitation, potential participants were informed of the objectives of the study, confidentiality of their responses and assured that they were free to withdraw from the study at any point in time. Once agreed to participate in the study, all participants provided written informed consent prior to the start of the interview. All patient responses remain confidential and the data is being stored securely in a password protected computer with access to the lead author (TMM) only. Data will be discarded 5 years after all the results of the study have been published, in accordance to institutional policies.

## 3 Results

### 3.1 Sample size, response rate and patient characteristics

**FIGURE 1:**
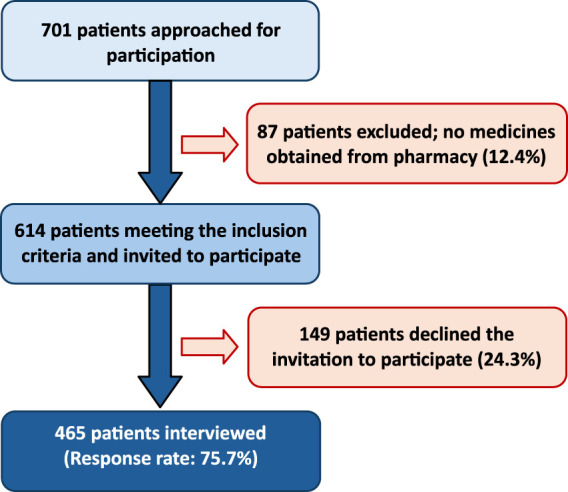
Patient flow.

A total of 701 patients were approached during the study, of whom 87 patients were not carrying any medication in their pharmacy or medicine bags and 149 patients declined the invitation to participate, which resulted in a final sample of 465 patients who were interviewed. Figure 1 illustrates the flow of patient recruitment and participation in this study.

The majority of interviewed patients were exiting from independent pharmacies (269; 57.8%), with the remainder exiting from either franchise (24.9%) or chain pharmacies (17.2%), which aligned with the distribution of different pharmacy categories in this rural province (Table 1).

The mean (39.7 years; SD: 13.0) and median (39 years; IOR: 18) ages of interviewed patients were similar, with equal distribution between males and females (Supplementary Table S5). Using the patient’s preferred language of communication, most interviews were conducted in English (45.8%). The interviews conducted in the other three languages were principally undertaken in Sepedi (28.6% of the total number of interviewed patients). The vast majority of interviewed patients (87.5%) had a qualification in addition to having completed secondary school (Supplementary Table S5).

### 3.2 Medicine items dispensed including antibiotics and/or purchased by patients from community pharmacies

Among the 465 interviewed patients, 127 (27.3%) were dispensed medicines with a prescription. Further details regarding the medicines dispensed with or without a prescription can be found in Maluleke et al. (2025c).

More than half (54.4%; 253/465) of the patients interviewed were dispensed at least one antibiotic, of whom only 54 patients (21.3%; 54/253) received these following a prescription. Of the 199 patients who received an antibiotic without a prescription, the majority (70.9%; 141/199) were for adults while 29.1% (58/199) were destined for children.

The majority of patients who were dispensed antibiotics without a prescription (n = 199) were dispensed these from independent (74.4%) and franchise (25.6%) pharmacies, with no chain pharmacy dispensing antibiotics without a prescription (Supplementary Table S6).

### 3.3 Conditions for which patients sought treatment and received an antibiotic

STIs were the most common infectious disease mentioned by 45.8% (116/253) of exiting patients for which they sought treatment from either a PHC clinic or community pharmacy, and received an antibiotic, either on prescription or without a prescription (Table 2). Notably, 99.1% (115/116) of patients who received STI-related antibiotics obtained their antibiotics without a prescription.

**TABLE 2:** Patients dispensed an antibiotic item with or without a prescription distributed by the perceived conditions for which patients sought treatment.

Condition for which treatment was sought	Number (%) of patients receiving an antibiotic*		Total number (%)**
	With a prescription	Without a prescription	
Sexually transmitted infection	1 (0.9)	115 (99.1)	116 (45.8)
Skin and soft tissue infection	8 (13.1)	53 (86.9)	61 (24.1)
Upper respiratory tract infection	32 (91.4)	3 (8.6)	35 (13.8)
Urinary tract infection	9 (64.3)	5 (35.7)	14 (5.5)
Vaginal thrush	0 (0.0)	13 (100)	13 (5.1)
Other infections/conditions^a^	4 (28.6)	10 (71.4)	14 (5.7)
Total	54 (21.3)	199 (78.7)	253

NB: *Row percentages; **Column percentages.

^a^ Other infections/conditions included diarrhoea, toothache, *Helicobacter pylori*, dental infection and eye infection.

As shown in Table 2, URTIs were the most common infectious disease for which patients were prescribed antibiotics, accounting for 59.3% (32/54) of these patients, with very limited dispensing of antibiotics without a prescription for patients with URTIs (1.5%; 3/199). The difference between the conditions for which patients sought treatment, and whether they received an antibiotic with or without a prescription, was statistically significant (p < 0.001) with a large effect size (Cramer’s V = 0.813).

### 3.4 Frequency distribution of patients’ knowledge of and attitudes toward the use of antibiotics and AMR

**FIGURE 2:**
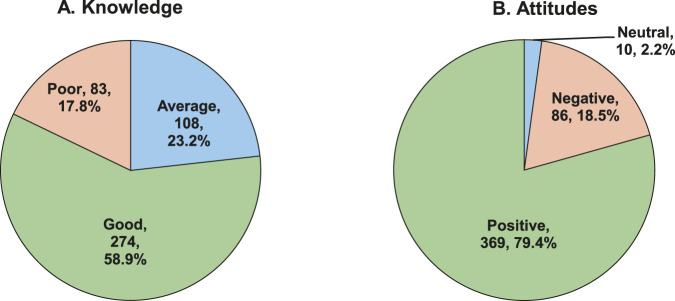
Categories of patients’ **(A)** knowledge of and **(B)** attitudes toward the use of antibiotics and AMR (n = 465).

Figures 2A,B respectively illustrate the proportion of knowledge and attitudes scores among participating patients when collapsed into categorical data. Overall, more than half (58.9%; 274/465) of the interviewed patients had good knowledge of the use of antibiotics and AMR, while most (79.4%; 369/465) had positive attitudes.

The frequency distribution of responses to the knowledge statements are shown in Table 3. Only 21.5% of the surveyed 465 patients knew that the statement ‘Antibiotic resistance occurs when your body becomes resistant’ was incorrect. However, the majority (76.3%; 355/465) correctly identified that with the overuse of antibiotics, organisms become resistant to the antibiotics. Of those patients who obtained their antibiotics without a prescription, 73.4% (146/199) responded correctly to this statement, with 78.6% (209/266) of the remaining patients similarly responding correctly.

**TABLE 3:** Patients’ knowledge about the use of antibiotics and AMR.

Knowledge statement^a^	Number (%) of patients (n = 465)		
	Correct answer	Incorrect answer	Don’t know
Antibiotic resistance occurs when your body becomes resistant (negative statement)	100 (21.5)	348 (74.8)	17 (3.7)
When people take too many antibiotics, the germs become resistant to them	355 (76.3)	13 (2.8)	97 (20.9)
Antibiotics can treat colds and coughs (negative statement)	301 (64.7)	164 (35.3)	0
Antibiotics are good for treating germs called bacteria	435 (93.5)	12 (2.6)	18 (3.9)
Taking antibiotics when not needed can lead to antibiotic	346 (74.4)	6 (1.3)	113 (24.3)
Completing the course is important, even when I feel better	442 (95.1)	5 (1.1)	18 (3.9)
I can share my antibiotics with someone else who is also ill or who needs them (negative statement)	435 (93.5)	7 (1.5)	23 (4.9)
In South Africa, community pharmacists are legally allowed to dispense antibiotics without a prescription (negative statement)	344 (74.0)	113 (24.3)	8 (1.7)
I must take antibiotics only when prescribed by a doctor or nurse	374 (80.4)	12 (2.6)	79 (17.0)
I can discard leftover antibiotics (negative statement)	360 (77.4)	23 (4.9)	82 (17.6)

^a^ Instruction for patients: Please select the best option, i.e., ‘True’, ‘False’ or ‘Don’t know’.

Among the 465 participating patients, just under two-thirds (64.7%; 301/465) correctly identified that antibiotics do not treat colds. The correct responses were similar among patients who were dispensed antibiotics without a prescription and the remainder at 63.3% (132/199) and 63.5% (169/266) respectively.

Table 4 shows the frequency distribution of responses to the five attitude statements. The vast majority of patients (94.8%; 441/465) supported the view that pharmacists should educate patients on the proper use of antibiotics, which was consistent for both group of patients. This was 94.0% (187/199) among patients who received antibiotics without a prescription *versus* 95.5% (254/266) among the remaining patients. More than 80% of patients agreed that AMR is a community concern, that everyone should take responsibility for the use of antibiotics, and that the government and regulatory bodies are also responsible for addressing and preventing AMR.

**TABLE 4:** Patients’ attitudes toward the use of antibiotics and AMR.

Attitude statement^a^	Number (%) of patients (n = 465)		
	Positive attitude	Neutral attitude	Negative attitude
Pharmacists should educate patients on proper antibiotic use	441 (94.8)	0	24 (5.2)
Antibiotic resistance is something the community should be concerned about	375 (80.6)	90 (19.4)	0
Healthcare personnel are the only ones responsible for addressing and preventing antibiotic resistance (negative statement)	356 (76.6)	79 (17.0)	30 (6.5)
Everyone needs to take responsibility for using antibiotics	372 (80.0)	93 (20.0)	0
Government and regulatory bodies are also responsible for addressing and preventing antibiotic resistance	410 (88.2)	55 (11.8)	0

^a^ Instruction for patients: Please select the best option, i.e., ‘True’, ‘False’ or ‘Don’t know’.

In terms of the negative statement that HCPs are the only ones responsible for addressing and preventing AMR, only 23.4% (109/465) of surveyed patients were neutral or agreed to this (negative attitude), with similar attitudes expressed by patients who received antibiotics without a prescription (22.1%; 44/199) and the remainder (17.3%; 46/266).

Overall, there was strong agreement with key stewardship principles and that AMR is widely recognised as a shared concern, with pharmacists, communities and patients all seen as important actors. For all the attitude statements, there was no statistically significant differences (p > 0.05) between the group of patients who received antibiotics without a prescription and the remainder.

### 3.5 Knowledge and attitude scores of patients on the use of antibiotics and AMR

Overall knowledge scores ranged from 1/10 obtained by 1.1% (5/465) of patients, to 9/10 obtained by 58.9% (274/465) of patients, with a mean knowledge score of 7.5 (SD: 2.3) and a median of 9.0 (IQR: 3). Attitude scores ranged from 0/5, which occurred among 1.9% (9/465) of patients, to 5/5, which was obtained by 76.3% (355/465) of patients, with a mean attitude score of 4.5 (SD: 1.5) and a median of 5.

Table 5 shows the mean difference in knowledge and attitude scores between patients who received antibiotics without a prescription compared with the others. There was no statistically significant difference between the two groups in terms of knowledge (p = 0.501) or attitudes (p = 0.772).

**TABLE 5:** Comparison of mean knowledge and attitude scores stratified by patients who received antibiotics without a prescription and those who received medicines, including antibiotics, on prescription or OTC.

​	Patients’ knowledge scores		Patients’ attitude scores	
	Received medicines, including antibiotics on prescription or OTC	Received an antibiotic without a prescription	Received medicines, including antibiotics on prescription or OTC	Received an antibiotic without a prescription
n	266	199	266	199
Mean (SD)	7.57 (2.21)	7.43 (2.38)	4.18 (1.57)	4.23 (1.51)
Mean difference	0.1443		−0.0419	
95% CI	−0.276; 0.565		−0.326; 0.242	
p-value	0.501		0.772	

### 3.6 Association between receiving an antibiotic and patients’ knowledge of, and attitudes toward, the use of antibiotics and AMR

Overall, over half (55.7%; 151/253) of the surveyed patients who received an antibiotic from a community pharmacy had good knowledge regarding the use of antibiotics and AMR, with the majority (81.4%; 206/253) showing positive attitudes. Patients with good knowledge were more likely to obtain their antibiotics without a prescription compared to those who were dispensed their medicines with a prescription or purchasing OTC medicines, and have lower average/poor knowledge scores. However, this difference between the two patient groups was not statistically significant (p = 0.820) (see Supplementary Table S7).

Whilst not statistically significant (p = 0.318), patients with positive attitudes were 1.689 times more likely to obtain their antibiotics with a prescription compared to those with neutral/negative attitudes (see Supplementary Table S7).

### 3.7 Rationale for purchasing antibiotics without a prescription

Patients who obtained their antibiotics without a prescription provided a number of reasons for their request. Among the 199 patients who were dispensed an antibiotic without a prescription, the most frequently cited reason for obtaining these was prior use of the same antibiotic (56.8%; 113/199) with more than two-thirds of these patients having good knowledge (68.1%; 77/113) and the majority having positive attitudes (83.2; 94/113).

Other notable reasons for purchasing antibiotics without a prescription included long waiting times at PHC clinics (15.6%; 31/199) and financial constraints (6.0%; 12/199), with the latter potentially associated with the costs and loss of income incurred when travelling to PHC clinics and currently limited finances. However, potentially acknowledging the costs of any antibiotics dispensed. Table 6 provides further details on the reasons for purchasing antibiotics without a prescription in this study, distributed by patients’ knowledge and attitudes.

**TABLE 6:** Reasons for obtaining an antibiotic without a prescription distributed by knowledge of and attitudes toward antibiotic use and AMR.

Reason for requesting an antibiotic without a prescription	Knowledge			Attitudes		
	Good*	Average/Poor*	Total**	Positive*	Neutral/Negative*	Total**
Used same antibiotic before	77 (68.1)	36 (31.9)	113 (56.8)	94 (83.2)	19 (16.8)	113 (56.8)
Long waiting time	18 (58.1)	13 (41.9)	31 (15.6)	24 (77.4)	7 (22.6)	31 (15.6)
No money	6 (50.0)	6 (50.0)	12 (6.0)	9 (75.0)	3 (25.0)	12 (6.0)
No pertinent antibiotics at the clinic	5 (45.5)	6 (54.5)	11 (5.5)	7 (63.6)	4 (36.4)	11 (5.5)
Pharmacist recommended	3 (33.3)	6 (66.7)	9 (4.5)	7 (77.8)	2 (22.2)	9 (4.5)
Too cheap	4 (57.1)	3 (42.9)	7 (3.5)	5 (71.4)	2 (28.6)	7 (3.5)
Insisted on an antibiotic	1 (16.7)	5 (83.3)	6 (3.0)	5 (83.3)	1 (16.7)	6 (3.0)
Not applicable	2 (40.0)	3 (60.0)	5 (2.5)	4 (80.0)	1 (20.0)	5 (2.5)
Clinic too far	2 (66.7)	1 (33.3)	3 (1.5)	2 (66.7)	1 (33.3)	3 (1.5)
No doctor around	2 (100.0)	0	2 (1.0)	2 (100)	0	2 (1.0)
Total	120 (60.3)	79 (39.7)	199	159 (79.9)	40 (20.1)	199

NB: *Row percentages; **Column percentages.

### 3.8 Using a translated questionnaire to determine knowledge of, and attitudes toward, the use of antibiotics among patients

The use of a translated questionnaire was instrumental in facilitating patient understanding and engagement during data collection. Given the linguistic diversity among participating patients (Supplementary Table S6), using the translated version in the patient’s preferred language enabled clearer communication of key concepts between the interviewer and the patient regarding antibiotic use and AMR. This was particularly important for nuanced or technical items, which included those addressing the mechanisms of AMR or the role of individual behaviour in AMR development.

The benefit of using a translated questionnaire in the patients’ preferred language of communication was illustrated by no statistically significant differences in patients’ mean knowledge scores (p = 0.634) and mean attitude scores (p = 0.733) between the four languages used during the interviews as illustrated in Table 7.

**TABLE 7:** Mean knowledge and attitude scores distributed by the interview language.

​	Language	Number	Mean (SD)	p-value
Knowledge score (n = 465)	English	213	7.6 (2.2)	0.634
	Sepedi	133	7.4 (2.4)	
	Tshivenda	50	7.6 (2.2)	
	Xitsonga	69	7.4 (2.4)	
Attitude score (n = 465)	English	213	4.1 (1.6)	0.733
	Sepedi	133	4.2 (1.6)	
	Tshivenda	50	4.4 (1.3)	
	Xitsonga	69	4.3 (1.4)	

While the statement ‘Antibiotic resistance occurs when your body becomes resistant to antibiotics’ was technically inaccurate, the translated phrasing aligned with common patient interpretations and supported the broader future implications of the study, i.e., to emphasize the consequences of inappropriate antibiotic use. However, a ‘True’ response to this item was scored as ‘incorrect’, which adversely affected the overall knowledge score of this particular item.

Importantly, while the translated questionnaire improved accessibility and comprehension, no additional explanations or clarifications were provided by the data collectors during its administration. This preserved the integrity and consistency of the data collection process among the participating patients. The translated items were designed to reflect commonly understood language without altering the intended meaning of the original questions.

## 4 Discussion and next steps

This is, to the best of our knowledge, the first study to extensively interview patients in this rural province in South Africa in their preferred language concerning their attitudes towards antibiotics and AMR. As a result, help to provide a robust future direction to all key stakeholder groups in South Africa when combined with the findings from community pharmacy personnel in the same province (Maluleke et al., 2025a; Maluleke et al., 2025d). We believe this is important given, as mentioned, critical concerns surrounding current antibiotic utilisation patterns in South Africa, including appreciable prescribing of antibiotics for often self-limiting infections (Supplementary Table S3) and the increasing use of Watch antibiotics, alongside high AMR rates (Mendelson, 2022; Department of Health Republic of South Africa, 2024; Mendelson et al., 2025a). High levels of Watch antibiotics are also seen in other LMICs (Saleem et al., 2025; Li et al., 2023; Saleem et al, 2020; Rani et al, 2024; Song et al., 2025).

Encouragingly, in this study, we observed very little dispensing of antibiotics without a prescription for patients with suspected URTIs exiting community pharmacies (Maluleke et al., 2025c). This compares with an appreciable proportion of patients who stated they were prescribed antibiotics for their perceived URTIs, with 91.4% of all antibiotics for URTIs being prescribed by HCPs in PHC clinics. In fact, a perceived diagnosis of URTIs accounted for 59.3% of all antibiotics dispensed with a prescription. The findings from patients appears to align with findings from surveyed pharmacists and assistants in the same province where over 98% reported they routinely offered symptomatic relief first to patients with self-limiting infections such as influenza, colds and coughs rather than recommending antibiotics (Maluleke et al., 2025a). There were similar findings in the study of Mokwele et al. (2022), where no antibiotics were dispensed to simulated patients presenting to community pharmacies with URTIs, only UTIs suggestive of STIs (Mokwele et al., 2022). Alongside this, there was very good knowledge among community pharmacy personnel regarding antibiotics and their use, including their effectiveness, or lack of it, in a range of infections including self-limiting infections in a similar study with community pharmacy personnel in this province (Maluleke et al., 2025d). Community pharmacy personnel in this rural province in South Africa were also aware of the risks, including increasing AMR, associated with unnecessary dispensing of antibiotics without a prescription (Maluleke et al., 2025d). Having said this, Tesema et al (2025) documented that when antibiotics were being given to young children to treat their cough or fever in South Africa, over 68% of the antibiotics given came from unqualified sources. However, this included leftover antibiotics from friends or relatives (Tesema et al, 2025). This situation may reflect family members and friends stopping their prescribed antibiotics early when they felt better ready to give left-over antibiotics to young children when they became ill with a cough or fever. This was despite knowing that antibiotics may not work in this situation, and may contribute to AMR. However, knowing that HCPs readily prescribed antibiotics for URTIs when visiting PHC facilities (Chigome et al., 2023, Maluleke et al., 2025c). This is because the informal sector for antibiotics is currently very limited in South Africa versus other African countries (Maluleke et al, 2026); consequently, any informal use is likely to come from family members and friends.

We believe that overall our findings suggest that patients in this rural province were not putting undue pressure on community pharmacists and pharmacist assistants to dispense antibiotics without a prescription for self-limiting conditions such as URTIs. We believe this is helped by community pharmacy personnel typically suggesting symptomatic relief first to patients presenting with URTIs (Maluleke et al., 2025a). Alongside this, surveyed patients generally had favourable attitudes towards community pharmacists educating them about antibiotics and AMR, with an appreciable number of surveyed patients concerned with rising AMR rates in the country. In addition, surveyed patients generally had good knowledge that the overuse of antibiotics contributes to AMR, even among those purchasing antibiotics without a prescription. Nearly two-thirds of patients in this study also correctly identified that antibiotics do not treat colds. This contrasts with the situation seen among prescribers in South Africa (Supplementary Table S3), including in this study, as well as across LMICs (Supplementary Table S1), where patients often put pressure, perceived or otherwise, on prescribers to issue antibiotics for self-limiting conditions including viral infections. As a result, there is still a very important role for community pharmacy personnel in South Africa to educate patients about taking the full course of prescribed antibiotics, and re-enforcing the message that they should not be taken for self-limiting infections.

We are aware though there was a commonly held misconception that “Antibiotic resistance occurs when your body becomes resistant” in this study. It is important to note that this misconception may be partly rooted in the terminology and translation differences across different South African languages, where the distinction between resistance of bacteria versus the human body is less clear or translatable. Consequently, while overall knowledge scores were categorized as “good” for many participants, this particular item negatively affected scores. We believe this reflects more a linguistic and conceptual challenge than a lack of general awareness about AMR among patients. Overall, this nuance underscores the need to consider language effects and cultural understandings when interpreting knowledge assessments in multilingual contexts, as well as when HCPs speak with patients about antibiotics, AMR and AMS.

We saw though considerable dispensing of antibiotics without a prescription for patients believing they had STIs in this study, with only one patient (0.9%) seeking care from a PHC facility for this infection. The stated principal rationale for this situation was the familiarity with community pharmacy staff. There was a similar situation in the study of Mokwele et al. (2022) with greater dispensing of antibiotics without a prescription for simulated patients principally with UTIs suggestive of STIs, with no dispensing of antibiotics for simulated patients with URTIs (Mokwele et al., 2022). We were not surprised by the appreciable prevalence of perceived STIs in our study given high prevalence rates for STIs generally in South Africa versus other countries, including other LMICs (Frank et al., 2023; Harryparsad et al., 2023; Seloka et al., 2025). Overall, we believe, based on generally good knowledge of antibiotics and AMR among participating patients in our study, apart from a misunderstanding whether the body or germs become resistant to antibiotics, that this behaviour is more to do with possible embarrassment, stigma, and logistical barriers, including long distances, when patients visit PHC facilities rather than a lack of knowledge regarding antibiotics and AMR. This is because co-payments to see HCPs in PHC facilities appear not to be an issue in South Africa versus other African countries especially following the introduction of universal healthcare (Meyer et al., 2017; Saleem et al., 2025). In addition, there could well be issues of affordability with patients purchasing antibiotics without a prescription in rural South Africa *versus* having antibiotics provided free of charge in PHC facilities. This may help explain the very low, or no, purchasing of antibiotics without a prescription in the studies of Anstey Watkins et al. (2019) and Do et al. (2021). Having said this, this appears to be less of an issue in this study. Overall, we believe the findings suggests considerable issues to address going forward as to why patients with actual or suspected STIs appeared unwilling to seek care from PHC facilities for their infection. Other possible issues to explore could be whether patients are willing to wait for test results in PHC facilities to help confirm STIs or whether they would prefer to re-visit local community pharmacies later in the day for any test results. This is because these issues have been raised in previous studies in South Africa (Gigi et al., 2025; Jaya et al., 2025). Given the high prevalence rates of STIs in South Africa, we believe it is important that these patients are appropriately managed going forward including actively promoting preventative strategies.

We believe based on our findings that community pharmacists appear to be the first contact point for patients with UTIs suggestive of STIs, as well as STIs themselves, that they are well placed to offer advice to patients regarding both the prevention and management of UTIs and STIs. This has worked well in other LMICs, although there have been concerns unless community pharmacy personnel are adequately trained (Garcia et al., 2003; Khan et al., 2006; Kwena et al., 2008; Lan et al., 2014; Agardh et al., 2017; Kuguyo et al., 2025). Consequently, we believe this development should be explored further in South Africa especially as the patients in this study stated they were happy for community pharmacy personnel to educate them on antibiotics and AMR. This increasing role for community pharmacists builds on successful examples in other countries, including other LMICs, where community pharmacists have become critical members of primary care teams (Marfo and Owusu-Daaku, 2017; Maluleke et al., 2025b; Hedima and Okoro, 2025). This includes improving the management of patients with both infectious and non-infectious diseases (Marfo and Owusu-Daaku, 2017; Mahdavi and Esmaily, 2021; Lambert et al., 2022; Khan et al., 2025; Maluleke et al., 2025b), including successfully diagnosing and dispensing antibiotics for adult patients presenting with UTIs (Jebara et al., 2018; Sanyal et al., 2019; Wu et al., 2021; Maluleke et al., 2025b). Community pharmacists also provided a vital service during the recent COVID-19 pandemic educating patients and suggesting treatment options to reduce their symptoms as well as reduce the spread of the virus (Alshahrani, 2020; Cadogan and Hughes, 2021; Kibuule et al., 2021; Mohamed et al., 2022). As a result, we believe there is now a strong rationale for the authorities in South Africa to consider extending the role and responsibilities of community pharmacists in South Africa. This involves becoming actively involve in the prevention and management of target infectious diseases, especially given the current shortages of physicians in PHC facilities, including in rural areas, prevailing unemployment among community pharmacists, and the current critical situation regarding AMR (Ntuli and Maboya, 2017; Chigome et al., 2023; Nyamuzihwa et al., 2023; Bateman, 2024; Mendelson et al., 2025; Maluleke et al., 2025b). These potential developments will mean upskilling community pharmacists, which could well include improving their skills to diagnosis and appropriately manage patients with STIs, alongside legislative reform to expand pharmacists’ scope of practice (Hedima and Okoro, 2025; Maluleke et al., 2025b; Mendelson et al., 2025b). Accompanying this, extending the number of universities in South Africa providing primary care drug therapy courses beyond the current single South African University (Tromp et al., 2023; Maluleke et al., 2025b). We are aware, there could be objections from prescribers across South Africa to such developments (Mendelson et al., 2025b). Possible objections could be reduced by community pharmacy personnel working alongside prescribers in the future to audit and improve their antibiotic prescribing habits (Marfo and Owusu-Daaku, 2017; Lambert et al., 2025; Van Hecke et al., 2025; Zerbinato et al., 2025). As a result, we believe this suggestion should not be viewed as confrontation, more collaboration, especially given ongoing pressures and issues with HCPs managing diseases, including infectious diseases, in PHC facilities across South Africa (Ntuli and Maboya, 2017; Stewart et al., 2018; Bateman, 2024). Once implemented, there needs to be ongoing research to assess and monitor the impact of these changes on subsequent patient care and antibiotic use in the community alongside any potential changes in AMR rates (Table 8).

**TABLE 8:** Policy recommendations for key stakeholder groups to strengthen community pharmacy’s role in antimicrobial stewardship in South Africa.

Strategic area	Recommendation	Rationale
Legislative reform	Update current legislature to expand the scope of practice to allow trained community pharmacists to manage agreed infections with a selected list of antibiotics that can be dispensed for agreed infections including UTIs and STIs	• Builds on international models to enhance the role of community pharmacists in PHCs given current physician shortages in the public healthcare system in the country • In addition, improving access to effective care in rural areas where there are current barriers and challenges
Training and certification	• Implement a structured training programme mandated and agreed by all key stakeholder groups going forward including expanding the current Primary Care Drug Therapy Pharmacists programme • This builds on the current Pharmacists’ programmes, and will mean expanding the list of Universities offering accredited programmes (currently just one) • Training also includes making sure community pharmacy personnel are aware of the importance of counselling patients regarding antibiotics and AMR in their own language where possible as well as making sure patients take the full course of antibiotics prescribed and not share any leftover antibiotics	• Ensures safe, evidence-based diagnosis and the dispensing of antibiotics for agreed conditions including as UTIs when current laws are changed • This builds on the existing trust between community pharmacists and patients, and the fact that patients typically visit community pharmacists for the management of UTIs suggestive of STIs and STIs first ahead of HCPs at PHC facilities in South Africa
Collaborative antimicrobial stewardship	Establish and grow pharmacist–prescriber partnerships for audit and feedback of current antibiotic prescribing habits to improve future prescribing and care of patients/reduce AMR	Reduces prescriber barriers and challenges to an increased role of community pharmacists with managing an agreed list of infectious diseases in the community as well as promoting shared responsibility for antibiotic use given current concerns
Monitoring and infrastructure	Develop systems to improve the tracking of antibiotic dispensing patterns among community pharmacies to improve their future use as part of any expanded programmes among community pharmacists and ASPs	Enables accountability, supports data-driven policies, and identifies trends in AMR-related behaviour that can be used to instigate future ASPs
Public health integration	• Position pharmacists as educators and first-line advisors on the appropriate use of antibiotics -including STI prevention and management - as well as the prevention of AMR - and the resultant implications with improving future patient care • This builds on the critical role of community pharmacists during the recent COVID-19 pandemic • Where possible, community pharmacists should counsel patients in their preferred language given the experience in this and other studies among LMICs	• Leverage current patient trust with community pharmacists and their accessibility, especially in rural provinces • Such activities will help support national AMR containment strategies given current concerns
Workforce development	• Address current unemployment among community pharmacists by improving their role to reduce current high AMR levels in primary care • Upskill the activities and knowledge of community pharmacists to improve their role and attractiveness across all provinces, especially rural provinces	Such activities align with national employment goals and strengthen primary healthcare capacity in South Africa, which is currently struggling to meet increasing demand for both the management of infectious and non-infectious diseases

NB: ASP, antimicrobial stewardship programme; AMR, antimicrobial resistance; HCPs, Healthcare professionals; PHC, primary healthcare; = Sexually transmitted infections; URTIs, Upper respiratory tract Infections; UTIs, Urinary tract infections.

Other policy recommendations to improve future antibiotic use in primary care in South Africa are also discussed in Table 8. These suggestions may also be applicable to other countries in sub-Saharan Africa and wider facing similar challenges (Maluleke et al., 2025b). We believe the combined activities in Table 8 are potentially more important, and could be appreciably more productive, than the Government in South Africa spending considerable time and resources educating patients further on the appropriate use of antibiotics. This is especially the case for the management of patients with STIs where we believe new approaches are needed in South Africa to improve subsequent patient care and reduce future prevalence rates (Johnson et al., 2025).

The experience of the data collectors in this study also highlights the importance of careful pretesting and cognitive validation of the translated tools during the pilot phases, especially when assessing the technical knowledge of patients in diverse populations. Future studies may consider refining such items to balance linguistic accessibility with scientific accuracy. In addition, researchers should remain cognisant that knowledge scores provided may well be adversely affected if the language of the questionnaire is not the patient’s first or preferred language, and this is not taken into consideration during questioning.

We are aware of a number of limitations with this study. Firstly, we are aware that one of the reasons patients requested antibiotics without a prescription could be their prior experience using the same antibiotic. However, we did not ascertain whether any previous antibiotic use was based on a valid prescription or self-purchasing. We are also aware that some patients may well have been asked by nurses in PHC facilities to purchase specific antibiotics from community pharmacies as no stocks were available in the PHC facility at that time and they were unable to issue external prescriptions. These patients were subsequently classified as paying for their antibiotics, i.e., without a prescription, for ease of analysis. However, we believe the numbers are likely to be small as there was limited dispensing of antibiotics without a prescription for patients with URTIs in our study compared to considerable prescribing for this indication, which suggests adequate stock levels for a range of antibiotics among PHCs in this province. We also did not ask patients whether they were just visiting this province or not when interviewed. However, in view of the local dynamics in this rural province, we believe this would only be a small number of patients at best. Alongside this, we did not discuss patients’ symptoms before classifying them, relying on feedback from the patients themselves. It should be noted that the study relied on patient self-reporting of the indication for which antibiotics were dispensed, representing perceived rather than clinically verified diagnosis. This approach introduces potential misclassification bias, especially relevant for overlapping or related conditions such as STIs and UTIs. Patients may have misinterpreted or inaccurately recalled their condition, leading to challenges in precisely categorizing antibiotic use by infection type. However, despite this, we believe the data provide valuable insights into patients’ perceptions and behaviours, which are important drivers of antibioitc use, including requesting antibiotics without a prescription, and stewardship efforts. In addition, we did not discuss patients’ employment status during questioning. Finally, we are also aware that we conducted this study in only one rural province in South Africa for the reasons specified. In view of this, the findings from this study may not be fully applicable to other settings in South Africa or other LMICs. However, despite these various limitations, we believe that overall our findings are robust providing appreciable insights that can be taken forward by all key stakeholders in South Africa and wider to improve future antibiotic use in primary care including an extended role for community pharmacists.

## 5 Conclusion

In conclusion, we believe this study offers a detailed examination of current antibiotic prescribing and dispensing patterns in this rural South African province. In addition, the current knowledge of patients visiting community pharmacists and being dispensed medicines, as well as their knowledge of key aspects regarding antibiotics, AMR and AMS. There were encouraging signs regarding the knowledge of patients concerning these important areas, with no obvious differences between patients dispensed antibiotics without a prescription or not. This has important ramifications going forward suggesting that valuable resources could be better spent in South Africa training and increasing the number of community pharmacists, including for preventing and managing STIs, versus expensive patient educational programmes.

Finally, we believe the findings provide a robust argument for trained community pharmacists in South Africa to be able to dispense agreed antibiotics without a prescription for patients with suspected UTIs and STIs, building on successful experiences in other countries. There is also a need to address the rationale behind current high rates of dispensing of antibiotics without a prescription for patients with STIs and potential ways forward to improve patient care. This could lead to the instigation of pertinent STI prevention strategies involving community pharmacists and pharmacist assistants, building on published studies in this area. Such activities can be combined with increased interactions with prescribers as part of ASPs to improve antibiotic utilisation in primary care in South Africa, and thereby reduce AMR.

## Data Availability

The original contributions presented in the study are included in the article/Supplementary Material, further inquiries can be directed to the corresponding author.
