# Supplementary material for: Knowledge and attitudes towards antibiotics and antimicrobial resistance among patients in rural South Africa and the implications for future policies
Source: Front Pharmacol. 2026 Apr 10;17:1756239. doi: 10.3389/fphar.2026.1756239 (PMC13105889; doi:10.3389/fphar.2026.1756239)
Supplement: Supplementary file 1 [file Supplementaryfile1.docx]

Supplementary Material

**Supplementary Table S1 -** Examples of the influence of patients on subsequent antibiotic use across LMICs including African countries

| **Country, authors and year** | **Key findings of interest** |
| --- | --- |
| Ethiopia - Erku et al., 2018 (1) | Perceived financial benefits alongside high expectations from patients and their demands were seen as principal drivers for high rates of dispensing of antibiotics without a prescription in the country |
| Ethiopia - Altaye et al., 2024 (2) | Pressure on prescribers from patients to prescribe antibiotics is exacerbated by limited knowledge and awareness regarding antibiotics coupled with a lack of respect for prescribers |
| India - Nair et al., 2019 (3) | Patient demands were seen as a key reason why over 88% of physicians in PHC clinics surveyed in this study reported prescribing antibiotics for essentially viral infections |
| Jordan - Orubu et al., 2022 (4) | - Two thirds (65%) of prescribers in PHC clinics taking part in this study reported pressure to prescribe antibiotics based on a number of factors - Key factors included the patients’ condition (78%); perceived patient demand (60%); and the perceived need to provide patients with rapid relief (47%) |
| Kenya - Mekuria et al., 2019 (5) | Clinician and patient perceptions that antibiotics should be prescribed for patients with acute respiratory tract infections resulted in high rates of prescribing of antibiotics for these infectious diseases |
| Malawi - MacPherson et al., 2022 (6) | Limited time with each patient in healthcare centres coupled with fear of being criticised for not prescribing medicines including antibiotics resulted in high rates of prescribing of antibiotics in primary care including for essentially self-limiting conditions |
| Nepal - Acharya et al., 2021 (7) | In this study in Nepal, high rates of dispensing of antibiotics without a prescription were influenced by pressure from patients especially when patients specifically asked for ‘an antibiotic’  This was because customer satisfaction was a key motivating factor even when antibiotics were not warranted |
| Tanzania - Emgard et al., 2021 (8) | Most mothers expected healthcare professionals in the healthcare clinics to prescribed an antibiotic for their child with an infectious disease whatever the cause – even when the infection was viral in origin |

**Supplementary Table S2** - Knowledge and attitudes of patients and parents regarding antibiotics and AMR across LMICs including African countries

| **Country, authors and year** | **Knowledge/ attitudes of patients regarding antibiotics and AMR** |
| --- | --- |
| Bangladesh - Islam et al., 2024 (9) | - An appreciable proportion of surveyed parents had limited knowledge regarding antibiotics - 63% and 56% respectively of parents did not know that amoxicillin and azithromycin were antibiotics - 63% of surveyed parents believed that antibiotics could be used to treat colds and improve a fever combined with a cold - 27% of surveyed parents reused the same antibiotic for similar symptoms |
| Egypt - Alsayed et al., 2022 (10) | - 58.8% of surveyed patients believed antibiotics were effective against sore throats and 57.6% that they were effective against colds, coughs and nasal congestion - 48.3% believed antibiotics were effective against fevers and 39.4% that they were effective against viral infections generally |
| Ethiopia - Muhummed et al., 2024 (11) | - 89.5% of surveyed patients believed that antibiotics can treat watery diarrhoea, - 71% believed antibiotics could treat a fever - 68.2% believed antibiotics could to treat a common cold with 57.7% believing they could treat general viral infections |
| Ghana - Vicar et al., 2023 (12) | - 77.0% of 600 household participants believed antibiotics can be used to cure influenza - 42.4% of households believed that antibiotics can be used to treat headaches/ coughs |
| Malawi - Machongo et al., 2022 (13) | - Caregivers of children under 5 typically self-medicated their children with antibiotics to treat coughs, sore throats and diarrhoea believing they were effective in this situation - Inappropriate self-medication with antibiotics was practiced through buying antibiotics without a prescription, using left-over antibiotics, and sharing of antibiotics |
| Nepal - Rijal et al., 2021 (14) | - 42.9% of surveyed patients believed a fever could be treated with antibiotics - 35.2% that antibiotics can be used to treat colds/ influenza - 17.3% believed antibiotics can be used to treat a sore throat |
| Nigeria - Isah et al., 2023 (15) | - 50.9% of surveyed patients believed that antibiotics can relieve fever and pain - 27.9% that using leftover antibiotics was permissible for treating another cold or similar symptoms - 22.1% believed antibiotics can cure their cold/sore throat more quickly |
| Pakistan - Gillani et al., 2021 (16) | - 35.4% of members of the public believing antibiotics could cure viral infections - Only 47.8% disagreed that antibiotics are effective against colds and influenza - Only 46.3% agreed that it is not necessary to treat a cold with antibiotics |
| Zambia - Kampamba et al., 2024 (17) | - 58.2% of surveyed patients stated they had taken an antibiotic for a common cold - 70.5% stated they had used leftover antibiotics from previous courses - 74.0% stated they had bought their antibiotics without a prescription |

**Supplementary Table S3** – Ongoing concerns with the prescribing of antibiotics in primary care in South Africa

| **Author and year** | **Objectives and methods** | **Key results** |
| --- | --- | --- |
| Farley et al., 2018 (18) | - Research attitudes, knowledge and practices concerning antibiotics and antibiotic resistance among prescribers in primary care - Cross-sectional survey - 264 prescribers completed the survey - 98.3% physicians with most (84.8%) practising in the private sector | - 95.8% of prescribers believed antibiotic resistance is a major problem in South Africa - 87.5% expressed a desire for additional education regarding the appropriate use of antibiotic with 96.2% seeking data on local antibiotic resistance patterns, with prescribers interested in updated STGs in various formats to improve their future prescribing - However, 66.5% felt pressure from patients to prescribe antibiotics for their infectious disease irrespective whether antibiotics were needed |
| Gasson et al., 2018 (19) | - Assess current antibiotic prescribing habits among prescribers in PHCs and against national STGs - Retrospective review of antibiotic prescribing habits/ ssessing potential reasons for non-adherence - 654 patient records reviewed | - 68.7% of attending patients were prescribed an antibiotic, with only 45.1% adherence to current STGs - Principal reasons for non-adherence to STGs included :   - undocumented diagnoses (30.5% of prescriptions)   - antibiotics not required - including for self-limiting infections (21.6%)   - incorrect doses prescribed (12.9%), incorrect duration of antibiotic therapy (9.5%)   - incorrect antibiotic for the infectious disease (1.5%) |
| Truter and Knoesen, 2018 (20) | - Assess primary care antibiotic prescribing habits using a self-administered questionnaire - 16 community pharmacists participated | - 81.3% of surveyed community pharmacists believed physicians were over-prescribing antibiotic - including for viral infections, potentially enhanced by patient pressure - Amoxicillin /co-amoxiclav were the most prescribed antibiotics - - Surveyed community pharmacists believed sinusitis and URTIs were the most common infections where antibiotics were inappropriately prescribed |
| van Hecke et al., 2019 (21) | - Determine perceptions of PHC physicians regarding their prescribing of antibiotics for patients presenting with acute coughs and UTIs plus experiences with point-of-care testing - Qualitative interviews among 23 prescribers | - Antibiotic prescribing decisions were typically influenced by (1) clinical assessment of presenting patients, (2) patients’ comorbidities and (3) their perceptions regarding patient expectations - Observed difficulties in communications between prescribers and patients – often hampered efforts by physicians to explain non-antibiotic management strategies to patients - Physicians typically positive towards point-of-care testing; however, concerns with current resources and workflow issues |
| Balliram et al., 2021 (22) | - Assess knowledge, attitudes and practices of doctors, pharmacists and nurses regarding antimicrobials, AMR and AMS - National online survey conducted | - 96.4% of doctors believed AMR was a severe global threat, with 96.6% believing AMR is a significant problem in South Africa. However, only 37.70% felt ≤ 50% confident in their knowledge regarding antimicrobials, AMR and AMS - 94.9% believed antibiotics were not effective against viral infections (vs. e.g. 75.3% for nurses) and 99.1% that common colds are caused by viruses (vs. e.g. 90.2% nurses). - 91.61% of participating professionals believed the overuse of antimicrobials was the greatest contributor to AMR, followed by patient pressure (75.26%) |
| Alabi et al, 2022 (23) | - Assess the appropriateness of antibiotic prescribing among practicing GPs in the private sector - Analysis of antibiotic prescriptions (188,141) among 174,889 patient records - Appropriateness based on the ICD-10 classification/ whether an antibiotic was warranted | - 92.9% of assessed patients were prescribed one antibiotic, with 7.1 % prescribed two or more antibiotics - 46.1% of all diagnoses made included diseases of the respiratory system, with only 8.8% of all antibiotic prescriptions deemed as appropriate and 32.0% as potentially appropriate - 45.4% of antibiotic prescriptions were deemed inappropriate and 13.8% could not be assessed due to a lack of specific codes/ contained unlisted codes/ or contained unclear descriptions in the prescription |
| Guma et al., 2022 (24) | - Assess current antibiotic empiric prescribing habits among private GPs among patients attending with ARIs - and associated key factors - Semi-structured web-based questionnaire was used - 209 GPs took part | - 55.5% admitted prescribing antibiotics empirically for ARIs more than 70% of the time - primarily for symptom relief and prevention of complications - GPs with more experience and working alone were slightly less likely to prescribe antibiotics empirically - Key factors associated with empiric antibiotic prescribing were diagnostic uncertainty, workload/time pressures and the use of a formulary |
| Lagarde and Blaauw, 2023 (25) | - Assess prescribing practices for young and healthy simulated patients visiting PHCs with viral bronchitis - Alongside this, 125 prescribers were interviewed using a structured questionnaire | - Antibiotics were recommended in 72.6% of consultations, higher in the public sector (78.4%) vs. private sector (66.7%) - - exacerbated by perceived patient pressure - This was despite 84% of prescribers knowing the patient was likely to have a viral rather than bacterial infection (88% in the private sector vs. 77% in the public sector) and 58% knowing antibiotics would not hasten recovery - 47% of prescribers in public PHCs thought patients would not come back if no antibiotic was prescribed – higher in the private sector at 72% (p=0.008) - despite no patient demanding these |
| Chigome et al, 2025 (26) | - Point prevalence survey among PHCs in 2 Provinces in South Africa and repeated - Part of a larger global study - 615 patients were evaluated | - Most common symptoms where antibiotics were prescribed included a genital discharge (21.8%), painful urination (18.4%), acute cough (17.7%), and a sore throat (13.5%). Patients could potentially have more than one symptom - At least one antibiotic was prescribed for 87.0% of attending patients, with 46.6% of prescribed antibiotics from the Watch group |
| Maluleke et al, 2025 (27) | - Assess antibiotic dispensing practices in a rural Province - 75.7% (128/169) of operational pharmacies took part in this questionnaire-based study | - Antibiotics accounted for 47.9% of all medicines dispensed, with penicillins the most dispensed antibiotic (41.1%) - 47.2% of antibiotics dispensed included cephalosporins, macrolides and fluoroquinolones – typically Watch antibiotics, with STIs (33.5%) and URTIs (25.8%) the most frequent believed indication for antibiotics |
| Maluleke et al, 2025 (28) | - Assess antibiotic usage patterns among patients in a rural province - 465 patients were interviewed as they left community pharmacies with a medicine | - 54.4% of patients were dispensed at least one antibiotic - STIs were the most common infectious disease for which an antibiotic was dispensed (60.1%), with 99.6% dispensed without a prescription - URTIs were the most common infection where antibiotics were dispensed with a prescription (60.0%), with little dispensing without a prescription (7.1%). |

NB: ABR = Antibiotic Resistance; AMR = Antimicrobial Resistance; AMS = Antimicrobial Stewardship; ARI = Acute Respiratory Infection; AWaRe = Access, Watch and Reserve [17]; EML = Essential Medicines List; GPs = General Practitioners; PHCs = Primary Healthcare Clinics; RTIs = Respiratory Tract Infections; SPs = Simulated Patients; STGs = Standard Treatment guidelines; STIs = Sexually Transmitted Infections; URTIs = Upper Respiratory Tract Infections; UTIs = Urinary Tract Infection

**Supplementary Table S4** – Patient Questionnaire

**Date: __________ Participant no: _______**

**Greet the patient and invite them to participate in the survey. Provide the patient with the participant information sheet or read it for them. Upon agreement to participate in the survey, obtain signed informed consent.**

| G 1. Age | | | | _____ years | | | | Prefer not to disclose age | | | | |
| --- | --- | --- | --- | --- | --- | --- | --- | --- | --- | --- | --- | --- |
| 2. Biological sex assigned at birth | | | | Male | | | Female | | | | Prefer not to answer | |
| **3. Home language** | Xitsonga | Tshivenda | | | Sepedi | | English | | Other (Specify) | | | |
| **4. Educational level** | No education | | Primary school completed | | | High school completed | | | | ABET  certificate | | College certificate |
|  | Diploma | | Bachelor’s degree | | | Honours degree | | | | Master’s degree | | Doctorate |

| **5. Were you dispensed or sold any medication at the pharmacy today?** | | | | | | | | | | | | Yes | | | | | No | | |
| --- | --- | --- | --- | --- | --- | --- | --- | --- | --- | --- | --- | --- | --- | --- | --- | --- | --- | --- | --- |
| **6. Did you have a prescription from a doctor?** | | | | | | | | | | | | Yes | | | | | No | | |
| **7. Does the medication include any antibiotics?** | | | | | | | | | | | Yes | | | | No | | | Don’t know | |
| **8. For what condition did you visit the pharmacy?** | | | | | | | | | | | | | | | | | | | |
| **How many items were dispensed/sold to you?** | | Yes | | No | | | ***Note: If no antibiotics were dispensed, end the interview and thank the patient for their time*** | | | | | | | | | | | | |
| **9. If antibiotics were dispensed, ask to have a look at the antibiotics dispensed and enter the details** | | | | | | **Antibiotics dispensed** | | | | | | | | **Other medication** | | | | | |
|  |  |  |  |  |  | 1. | | | | | | | | 1. | | | | | |
|  |  |  |  |  |  | 2. | | | | | | | | 2. | | | | | |
|  |  |  |  |  |  | 3. | | | | | | | | 3. | | | | | |
|  |  |  |  |  |  | 4. | | | | | | | | 4 | | | | | |
| **10. What were the antibiotics indicated for?** | URTI (Upper respiratory tract infection) | | | | | | |  | SSTI (Skin and soft tissue infection) | | | | | | | | | |  |
|  | STI (Sexually transmitted disease) | | | | | | |  | UTI (Urinary tract infection) | | | | | | | | | |  |
|  | Other (Please specify) | | | | | | | | | | | | | | | | | | |
| **11. Who were the antibiotics for?** | | | | | Adult | | | | Child | | | | | | | Both | | | |
| **12. If the medication is for a child under 12, are you the parent or guardian?** | | | | | Parent | | | Guardian | | | | | Other (specify) | | | | | | |
| **13. If the medication is for a child, how old are they?** | | | | | | | | | ____________ years/months | | | | | | | | | | |
| **14. If any antibiotics were obtained without a prescription, what were the reasons?** | | | Clinic too far | | | | | | | Long waiting times | | | | | | | | | |
|  |  |  | No money to see a doctor | | | | | | | Used the same antibiotics before | | | | | | | | | |
|  |  |  | Lack of knowledge | | | | | | | Don’t know | | | | | | | | | |
|  |  |  | Pharmacist recommended them | | | | | | | Patient insisted on an antibiotic | | | | | | | | | |
|  |  |  | Other (Please specify) | | | | | | | | | | | | | | | | |

**Thank the patient for their time and participation**

**Supplementary Table S5** - Sociodemographic characteristics of patients interviewed on exit from the different community pharmacy categories

| **Pharmacy category and sociodemographic characteristics** | | **Number (%) of participants (N=465)** |
| --- | --- | --- |
| **Pharmacy category** | Franchise | 116 (24.9) |
|  | Chain | 80 (17.2) |
| **Sex** | Male | 232 (49.9) |
|  | Female | 233 (50.1) |
| **Language** | English | 213 (45.8) |
|  | Sepedi | 133 (28.6) |
|  | Xitsonga | 69 (14.8) |
|  | Tshivenda | 50 (10.8) |
| **Education Level** | None | 2 (0.4) |
|  | Secondary School Completed | 56 (12.0) |
|  | ABET Certificate | 5 (1.1) |
|  | College Certificate | 76 (16.3) |
|  | Diploma | 145 (31.2) |
|  | Honours | 5 (1.1) |
|  | Bachelor’s Degree | 145 (31.2) |
|  | Master’s Degree | 27 (5.8) |
|  | Doctoral Degree | 4 (0.9) |

**Supplementary Table S6 -** Details of antibiotics who received an entibiotic with or without a prescription by pharmacy category

|  | **Number (%) of patients who received an antibiotic** | | |
| --- | --- | --- | --- |
| **Pharmacy category** | **With a prescription*** | **Without a prescription*** | **Total**** |
| Independent pharmacies | 13 (8.1) | 148 (91.9) | 161 (63.9) |
| Franchise pharmacies | 14 (21.5) | 51 (78.5) | 65 (25.4) |
| Chain pharmacies | 27 (100) | 0 | 27 (10.7) |
| **Total** | **54 (21.3)** | **199 (78.7)** | **253** |

NB: *Row percentages; **Column percentages

**Supplementary Table S7** - Association between receiving an antibiotic and patients’ knowledge of, and attitudes toward, the use of antibiotics and AMR

| **Use of antibiotics and AMR** | | **Number (%) of patients who received an antibiotic*** | | **Total number (%)**** | **Odds Ratio  (95% CI)** | **p-value** |
| --- | --- | --- | --- | --- | --- | --- |
|  |  | **With a prescription** | **Without a prescription** |  |  |  |
| **Knowledge** | **Good** | 31 (20.5) | 120 (79.5) | 151 (59.7) | 0.887 (0.482; 1.632) | 0.820 |
|  | **Average/Poor** | 23 (22.5) | 79 (77.5) | 102 (40.3) |  |  |
| **Attitude** | **Positive** | 47 (22.8) | 159 (77.2) | 206 (81.4) | 1.689 (0.710; 4.018) | 0.318 |
|  | **Neutral/Negative** | 7 (14.9) | 40 (85.1) | 47 (18.6) |  |  |
| **Total** | | **54 (21.3)** | **199 (78.7)** | **253** |  | |

NB: *Row percentages; **Column percentages

**References – Supplementary Tables**

1. Erku DA, Aberra SY. Non-prescribed sale of antibiotics for acute childhood diarrhea and upper respiratory tract infection in community pharmacies: a 2 phase mixed-methods study. Antimicrob Resist Infect Control. 2018;7:92.

2. Altaye FW, Thupayagale-Tshweneagae G, Mfidi FH. Qualitative enquiry on factors affecting antibiotic prescribing at primary healthcare facilities in Addis Ababa, Ethiopia. Front Med (Lausanne). 2024;11:1308699.

3. Nair M, Tripathi S, Mazumdar S, Mahajan R, Harshana A, Pereira A, et al. Knowledge, attitudes, and practices related to antibiotic use in Paschim Bardhaman District: A survey of healthcare providers in West Bengal, India. PLoS One. 2019;14(5):e0217818.

4. Orubu ESF, Albeik S, Ching C, Hussein R, Mousa A, Horino M, et al. A Survey Assessing Antimicrobial Prescribing at United Nations Relief and Works Agency Primary Health Care Centers in Jordan. Am J Trop Med Hyg. 2022;107(2):474-83.

5. Mekuria LA, de Wit TF, Spieker N, Koech R, Nyarango R, Ndwiga S, et al. Analyzing data from the digital healthcare exchange platform for surveillance of antibiotic prescriptions in primary care in urban Kenya: A mixed-methods study. PLoS One. 2019;14(9):e0222651.

6. MacPherson EE, Reynolds J, Sanudi E, Nkaombe A, Phiri C, Mankhomwa J, et al. Understanding antimicrobial resistance through the lens of antibiotic vulnerabilities in primary health care in rural Malawi. Glob Public Health. 2021:1-17.

7. Acharya Y, Nepal P, Yang D, Karki K, Bajracharya D, Prentis T, et al. Economic and social drivers of antibiotic dispensing practices among community pharmacies in Nepal. Trop Med Int Health. 2021;26(5):557-71.

8. Emgård M, Mwangi R, Mayo C, Mshana E, Nkini G, Andersson R, et al. Tanzanian primary healthcare workers' experiences of antibiotic prescription and understanding of antibiotic resistance in common childhood infections: a qualitative phenomenographic study. Antimicrob Resist Infect Control. 2021;10(1):94.

9. Islam MW, Shahjahan M, Azad AK, Hossain MJ. Factors contributing to antibiotic misuse among parents of school-going children in Dhaka City, Bangladesh. Sci Rep. 2024;14(1):2318.

10. Alsayed AR, Darwish El Hajji F, Al-Najjar MAA, Abazid H, Al-Dulaimi A. Patterns of antibiotic use, knowledge, and perceptions among different population categories: A comprehensive study based in Arabic countries. Saudi Pharm J. 2022;30(3):317-28.

11. Muhummed AM, Alemu A, Maidane YO, Tschopp R, Hattendorf J, Vonaesch P, et al. Knowledge, Attitudes, and Practices of Rural Communities Regarding Antimicrobial Resistance and Climate Change in Adadle District, Somali Region, Ethiopia: A Mixed-Methods Study. Antibiotics . 2024;13(4).

12. Vicar EK, Walana W, Mbabila A, Darko GK, Opare-Asamoah K, Majeed SF, et al. Drivers of household antibiotic use in urban informal settlements in Northern Ghana: Implications for antimicrobial resistance control. Health Sci Rep. 2023;6(7):e1388.

13. Machongo RB, Mipando ALN. "I don't hesitate to use the left-over antibiotics for my child" practices and experiences with antibiotic use among caregivers of paediatric patients at Zomba central hospital in Malawi. BMC Pediatr. 2022;22(1):466.

14. Rijal KR, Banjara MR, Dhungel B, Kafle S, Gautam K, Ghimire B, et al. Use of antimicrobials and antimicrobial resistance in Nepal: a nationwide survey. Sci Rep. 2021;11(1):11554.

15. Isah A, Aina AB, Ben-Umeh KC, Onyekwum CA, Egbuemike CC, Ezechukwu CV, et al. Assessment of public knowledge and attitude toward antibiotics use and resistance: a community pharmacy-based survey. J Pharm Policy Pract. 2023;16(1):107.

16. Gillani AH, Chang J, Aslam F, Saeed A, Shukar S, Khanum F, et al. Public knowledge, attitude, and practice regarding antibiotics use in Punjab, Pakistan: a cross-sectional study. Expert Rev Anti Infect Ther. 2021;19(3):399-411.

17. Kampamba M, Hamaambo B, Hikaambo CN, Mwanza B, Bambala A, Mutenda M, et al. Evaluation of knowledge and practices on antibiotic use: a cross-sectional study on self-reported adherence to short-term antibiotic utilization among patients visiting level-1 hospitals in Lusaka, Zambia. JAC Antimicrob Resist. 2024;6(4):dlae120.

18. Farley E, Stewart A, Davies MA, Govind M, Van den Bergh D, Boyles TH. Antibiotic use and resistance: Knowledge, attitudes and perceptions among primary care prescribers in South Africa. S Afr Med J. 2018;108(9):763-71.

19. Gasson J, Blockman M, Willems B. Antibiotic prescribing practice and adherence to guidelines in primary care in the Cape Town Metro District, South Africa. S Afr Med J. 2018;108(4):304-10.

20. Truter I, Knoesen BC. Perceptions towards the prescribing of antibiotics by pharmacists and the use of antibiotics in primary care in South Africa. J Infect Dev Ctries. 2018;12(2):115-9.

21. van Hecke O, Butler C, Mendelson M, Tonkin-Crine S. Introducing new point-of-care tests for common infections in publicly funded clinics in South Africa: a qualitative study with primary care clinicians. BMJ Open. 2019;9(11):e029260.

22. Balliram R, Sibanda W, Essack SY. The knowledge, attitudes and practices of doctors, pharmacists and nurses on antimicrobials, antimicrobial resistance and antimicrobial stewardship in South Africa. S Afr J Infect Dis. 2021;36(1):262.

23. Alabi ME, Essack SY. Antibiotic prescribing amongst South African general practitioners in private practice: an analysis of a health insurance database. JAC Antimicrob Resist. 2022;4(5):dlac101.

24. Guma SP, Godman B, Campbell SM, Mahomed O. Determinants of the Empiric Use of Antibiotics by General practitioners in South Africa: Observational, Analytic, Cross-Sectional Study. Antibiotics. 2022;11(10):1423.

25. Lagarde M, Blaauw D. Levels and determinants of overprescribing of antibiotics in the public and private primary care sectors in South Africa. BMJ Glob Health. 2023;8(7).

26. Chigome MA, Vambe MS, Kganyago MK, Meyer PJ, Campbell PS, Godman PB, et al. Point prevalence surveys of acute infection presentation and antibiotic prescribing in selected primary healthcare facilities in North-West and Gauteng provinces of South Africa. International Journal of Infectious Diseases. 2025;152:107689.

27. Maluleke TM, Maluleke MT, Jelic AG, Campbell SM, Marković-Peković V, Schellack N, et al. Estimated extent of purchasing of antibiotics without a prescription from community pharmacies in a rural province in South Africa and the implications. Frontiers in Tropical Diseases. 2025;Volume 6 - 2025.

28. Maluleke TM, Maluleke MT, Ramdas N, Jelić AG, Kurdi A, Chigome A, et al. Prevalence and Associated Factors for Purchasing Antibiotics Without a Prescription Among Patients in Rural South Africa: Implications for Addressing Antimicrobial Resistance. Antibiotics. 2025;14(12):1273.
